# Leisure activities and leisure motivations of Chinese residents

**DOI:** 10.1371/journal.pone.0206740

**Published:** 2018-11-01

**Authors:** Meiai Chen, Shiyi Xue, Yan Shi

**Affiliations:** 1 School of Tourism & Health, Zhejiang A&F University, Hangzhou, Zhejiang, PR China; 2 Institute of Communication Studies, Communication University of China, Beijing, PR China; 3 School of Landscape Architecture, Zhejiang A&F University, Hangzhou, Zhejiang, PR China; University of Toronto, Rotman School, CANADA

## Abstract

453 residents of Hangzhou China (56.1% women, average age 34.68 with range of 18–65) were used to investigate leisure activities and leisure motivations. Demographic data such as gender, age, marital status, education level, and income were collected and analyzed. The results indicated the following: (a) Residents’ top three favorite activities were internet surfing, drinking tea and chatting, and traveling; (b) The Leisure Motivation Scale had a high reliability, with a Cronbach’s alpha of 0.93; (c) In general, residents’ intellectual, social, competence mastery, and stimulus avoidance scores all reached high levels; and (d) Significant differences among gender, marital status, and education level were found in leisure motivation. The findings are discussed in regard to the status quo of leisure behaviors and leisure motivations of Chinese residents.

## Introduction

Leisure motivation can be defined as a requirement, reason, or satisfaction that stimulates involvement in a leisure activity [1]; it is an intervening factor that stimulates a change from leisure motivated to leisure participated in residents. Intervention programming is the factor affecting leisure motivation among adolescents, and changes in self-reported motivation for leisure due to participation in Healthwise South Africa were investigated [2]. Leisure motivation had no significant effect on leisure satisfaction when leisure involvement was also in the model, but leisure motivation had a significant effect on leisure involvement [3]. Meanwhile, leisure involvement provides an effective means of segmenting physically active leisure participants, and self-efficacy and motivation for physical activity were predictably linked to involvement [4]. Motivations for leisure participation and its associations with psychological engagement were investigated, and the results showed that three of the five hypothesized motivators positively and significantly predicted greater psychological engagement [5].

Walker and his colleagues found that the type of self-construal each person has affects his or her emotions, cognitions, and motivations [6]. The researchers described what self-construal was and how it affected intrinsic motivation, and they reviewed some major theories that included intrinsic motivation, as well as discussing how these might simultaneously affect each other. Mannel believed that the authors’ paper contributed toward correcting the lack of use of these theories. Developing a leisure theory based on different cultures is important and can be used as a potential stimulant for future leisure theory research and development [7].

Culture significantly influences leisure motivation, as individual beliefs and attitudes partially rely on the social circumstances to which they belong. Social behavior is primarily guided by personal goals in individualistic cultures, while the goals of the collective have a dominant influence on forming behavior in collectivistic cultures [8]. Walker and Wang focused on the leisure motivations of Canadian and Mainland Chinese University students [9]. Their research results indicated that Canadian students had much more identification, interjected reward, and interjected punishment motivation than did Chinese students. In addition, Walker reported that separate hierarchical multiple regressions were performed on each motivation with culture entered first, followed by a block composed of four types of self-construal: vertical collectivism, horizontal collectivism, horizontal individualism, and vertical individualism [10]. Xu and Morgan performed a cross-cultural comparison of travel behavior from two different nations, namely, China and England [11]. They found that Chinese students believed it was more important to see famous sights and learn about other cultures and history, while the British were more concerned about having fun, socializing and enjoying the challenges of outdoor adventures. Liu and Walker examined the effects of micro (i.e., motivation and constraint) and macro (i.e., urbanization) factors on Chinese residents’ leisure-time physical activity (LTPA) [12]. They found that urbanization positively affected LTPA participation and the constraints to LTPA.

Additionally, the psychological values that motivate leisure and recreational cyclists were explored, and the major intrinsic motivational factors of cyclists were found to include competence mastery, solitude, exploration, physical challenge, adventure experiences, stimulus seeking, social encounters, and relaxation/escapism [13]. The leisure motivations of disabled people in Ghana were assessed, and the findings suggest that the motivations of people with visual and physical disabilities were fourfold, namely, competence mastery, social, intellectual, and stimulus avoidance [14]. The influence of perceived parental control and leisure restructuring ability on leisure motivation (amotivation and autonomous motivation) was explored by using samples of eighth-grade adolescents in the United States and South Africa. The results showed that the measurement model of the constructs was equivalent across the two samples, but the determinants of leisure motivation differed between the samples [15].

Leisure plays an important role in Chinese society. In recent years, substantial attention has been paid to the People’s Republic of China in the sphere of leisure studies. Therefore, leisure motivation has already become one of the important leisure studies in the Western world, but it is only slightly focused on in China, and its importance is often overlooked or ignored. Thus, this study aims to examine the relationship between leisure activities and leisure motivations of the residents in China, and the reported results will add to the limited research in the area of leisure motivation in collectivism nations.

## Method

### Ethics statement

An ethics approval was not required for this research as per Zhejiang Agriculture and Forestry University’s guidelines as well as regulations. However, an ethical approach is expected. For this research the oral consent of the participants was obtained after principles expressed in the Declaration of Helsinki. The study has no harm to participants, as the names of the residents have not been used in order to preserve anonymity, and the data were analyzed anonymously.

### Study methodologies

#### Leisure activities questionnaire

This questionnaire comprises four items, namely, leisure activities engaged in, weekly leisure time, leisure frequency, and weekly leisure spending. The respondents entered information related to the leisure activities they enjoy participating in frequently, the number of hours they spend on leisure each week, the number of occasions they participate in leisure each week, and their weekly leisure expenses.

#### Leisure Motivation Scale

To study leisure motivation, the Leisure Motivation Scale has been used in all kinds of settings. Beard and Ragheb developed a comprehensive list of 48 leisure motivations, which can be categorized into four subscales: intellectual, social, competence mastery, and stimulus avoidance. The shortened 32-item instrument was recommended for use in a research setting in which the time given for administration was a major consideration, and data could be combined across individuals [16]. As a result, this study utilized the shortened list of 32 items to measure the leisure motivation of residents in Hangzhou. Residents used a five-point Likert scale (1 = strongly disagree, 5 = strongly agree) to rate the 32 leisure motivation items.

### Study sample

Data for the current study were collected between 8 and 23 October 2016 in Hangzhou. A convenience sample of residents was selected and recruited to pilot in Zhejiang Library, West Lake, Shopping Mall (Intimes) and Zhejiang University; 25 residents refused to participate. After excluding the participants who did not meet the criteria, 453 residents remained.

### Participants

The majority of respondents (56.1%) were female. Half the residents were 25–35 years old. With regard to marital status, 47.2% of the responding residents were single and had relatively more time for leisure than did married residents. For education level, most of the residents had attained a college degree or above because Hangzhou is one of the immigrant cities in China, and its economic development level is very high. Regarding monthly income, more than half the residents have an income of over 3000 RMB.

### Data analysis

The statistics software package SPSS version 24.0 was used for all data entry and analyses. Data analysis consisted of three stages. First, frequencies were used to examine the residents’ current leisure situation. Second, the reliability of the Chinese version of the Leisure Motivation Scale (LMS) was examined. Third, independent samples t-tests and analysis of variance (ANOVA) were conducted on leisure motivation using personal background as the independent variable.

## Results

### Leisure activities engaged in

To examine leisure situations in Hangzhou, residents were asked what leisure activities they enjoy participating in frequently. Table 1 shows that the top three favorite leisure activities are internet surfing (56.1%), drinking tea and chatting (46.1%), and traveling (44.4%). The next four activities are watching films, TV, and cartoons (40.8%), taking a walk in the park (27.8%), visiting relatives and friends (17.0%), and sports (17.0%).

**Table 1:** Leisure activities engaged in.

Selected items		(%)
What leisure activities do you enjoy participating in frequently? (Choose three items)		
(a) internet surfing	56.1	
(b) drinking tea and chatting	46.1	
(c) traveling	44.4	
(d) watching films, TV, and cartoons	40.8	
(e) taking a walk in the park	27.8	
(f) visiting relatives and friends	17.0	
(g) sports	17.0	
(h) visiting (museum or celebrity house)	11.0	
(i) hobbies (e.g., painting, reading, photography and collections)	10.2	
(j) playing poker and mahjong	7.7	
(k) taking care of plants and pets	7.3	
(l) alcohol consumption	5.1	
(m) theatre	4.0	
(n) skincare, decorating the house	3.8	
(o) others	1.8	

Note: N = 453.

### Weekly leisure time, leisure frequency, and leisure spending

Table 2 shows the residents’ weekly leisure time and leisure frequency, and their weekly leisure expenses. The data from part 1 indicate that only 25.2% of the residents spend 7 hours or less on leisure each week, while more than 70% of residents spend more than 7 hours on leisure each week. The data from part 2 show that more than half the residents take part in leisure activities three times or less each week, while 31.8% of the residents take part in leisure activities 3–5 times each week. For weekly leisure spending, 61.5% of residents spend less than ¥200 RMB every week, and only 12.8% of residents spend more than ¥500 RMB on leisure activities every week.

**Table 2:** Weekly leisure time, leisure frequency, and leisure spending.

Selected Items	(%)
1. How many hours do you spend on leisure each week?	
(a) 7 hours or less	25.2
(b) 7–14 hours	35.3
(c) 14–21 hours	19.2
(d) 21 hours or more	20.3
2. On how many occasions do you participate in leisure each week?	
(a) three times or less	54.5
(b) 3–5 times	31.8
(c) 6–7 times	7.3
(d) 7 times or more	6.4
3. What are your weekly expenses on leisure (RMB)?	
(a) ¥100 or below	24.9
(b) ¥100–199	36.6
(c) ¥200–499	25.6
(d) ¥500 or more	12.8

Note: N = 453.

### Reliability of survey method

Cronbach’s alpha was used to estimate the reliability of the LMS in this research. Table 3 shows that Cronbach’s alpha coefficients were 0.88 in intellectual motivation, 0.85 in social motivation, 0.87 in competence mastery, and 0.81 in stimulus avoidance. The coefficient of the overall method was 0.93. The reliability coefficients of all the LMS dimensions exceeded 0.80; therefore, the dimensions can be regarded as very dependable.

**Table 3:** Reliability estimates for Leisure Motivation Scale (LMS).

Factors	Items	Cronbach’s Alpha
Intellectual Motivation	1,2,3,4,5,6,7,8	0.88
Social Motivation	9,10,11,12,13,14,15,16	0.85
Competence Mastery	17,18,19,20,21,22,23,24	0.87
Stimulus Avoidance	25,26,27,28,29,30,31,32	0.81

### Leisure Motivation Scale (LMS)

Data were collected by using the LMS, which is divided into four subscales, namely, Intellectual, Social, Competence Mastery and Stimulus Avoidance. The mean scores and standard deviations (SD) are shown in Table 4. The results indicated that these means are homogeneous.

**Table 4:** Means and standard deviations from the LMS.

Measure	M	SD	Minimum	Maximum	Rank
Intellectual Motivation	28.92	5.27	8	40	2
Social Motivation	27.89	5.08	8	40	4
Competence Mastery	28.72	5.03	8	40	3
Stimulus Avoidance	30.69	4.76	10	40	1

Note: N = 453. Scale ranged from 1–5.

### Inferential analysis

Independent sample t-tests were used to examine the differences in the four leisure motivations by gender. Table 5 shows the mean, SD, Levene’s Test of equality of variance, and results of leisure motivation by gender.

**Table 5:** Differences among the four dimensions of LMS by gender.

	Male		Female		Sig of Levene’s Test	t	d
	M	SD	M	SD			
IM	28.42	5.79	29.32	4.79	.037	-1.79	.16
SM	27.69	5.52	28.04	4.72	.098	-.71	.06
CM	27.99	5.04	29.29	4.96	.904	-2.74 **	.25
SA	29.82	4.74	31.38	4.68	.793	-3.49**	.33

Note. Male = 199, Female = 254.

*p < .05.

** *p* < .01.

***p < .000.

IM = intellectual motivation. SM = social motivation. CM = competence mastery. SA = stimulus avoidance.

There were significant differences in both competence mastery and stimulus avoidance between males and females (*p* < .01). The results showed that both males and females reported the highest scores in competence mastery and stimulus avoidance, but females had a much stronger desire to pursue peaceful and calm leisure elements than did males.

A one-way between groups ANOVA was used to examine the difference in the four leisure motivations by age. There was no significant difference found among leisure motivations by age. Post hoc analysis also indicated that there were no significant differences among age groups. Age did not seem to have any influence on leisure participation and leisure motivation. Table 6 shows the means and SD.

**Table 6:** Difference among the four dimensions of LMS by age.

	2003-		1993–2002		1983–1992		1973–1982		1963–1972		1963+	
	M	SD	M	SD	M	SD	M	SD	M	SD	M	SD
IM	29.00	9.21	29.76	5.22	28.87	5.20	28.41	5.11	26.31	4.46	26.69	5.25
SM	28.17	7.25	27.89	4.84	27.92	5.29	28.43	4.52	25.46	4.16	27.44	5.75
CM	30.00	8.76	29.76	5.21	28.53	5.05	28.78	4.19	27.15	4.32	27.63	4.92
SA	28.33	4.72	31.39	5.02	30.64	4.56	30.44	4.39	30.15	4.09	27.94	4.85

Note. N = 453.

*p < .05.

** *p* < .01.

***p < .000.

IM = intellectual motivation. SM = social motivation. CM = competence mastery. SA = stimulus avoidance.

ANOVA was used to examine the differences among the four leisure motivations by marital status. Table 7 shows the means, SD, ANOVA results, and Scheffé post hoc comparison.

**Table 7:** Difference among the four dimensions of LMS by marital status.

	(1) Single		(2) Married with Children		(3) Married with No Children		(4) Separated & Divorced		ANOVA F	Post Hoc
	M	SD	M	SD	M	SD	M	SD		
IM	29.68	5.43	28.24	4.29	28.14	5.14	33.25	7.80	4.12**	1>3 ^S^
SM	28.19	5.07	26.67	5.01	27.88	5.12	27.50	3.69	1.22	
CM	29.23	5.13	28.08	4.61	28.25	4.99	30.75	5.74	1.77	
SA	31.14	4.77	29.72	5.18	30.43	4.64	31.50	3.42	1.54	

Note. N = 453.

*p < .05.

** *p* < .01.

***p < .000.

^S^ = Scheffé.

IM = intellectual motivation. SM = social motivation. CM = competence mastery. SA = stimulus avoidance.

There was a significant difference found in intellectual motivation. Residents who are separated and divorced had the highest scores on the intellectual motivation subscale. The results indicated that single residents were more likely to show intellectual motivation.

One-way ANOVA was used to examine the differences among the four leisure motivations by education level. Table 8 shows the mean, SD, ANOVA results, and Scheffé post hoc comparison of leisure motivations by education level. There was a significant difference in social motivation among education levels. Residents with high education levels reported the lowest mean scores in social motivation.

**Table 8:** Differences among the four dimensions of LMS by education level.

	(1) Senior School or Lower		(2) Junior College		(3) Undergraduate College		(4) Master’s/PhD		ANOVA F	Post Hoc
	M	SD	M	SD	M	SD	M	SD		
IM	29.59	4.86	28.74	5.48	28.96	5.19	28.18	5.64	1.08	
SM	28.25	4.66	28.07	4.56	28.43	4.94	25.86	6.19	4.89**	1,2,3>4^S^
CM	29.25	4.55	28.66	5.39	28.73	5.24	28.04	4.64	.83	
SA	30.06	4.83	30.59	5.06	31.42	4.59	30.07	4.46	2.35	

Note. N = 453.

*p < .05.

** *p* < .01.

***p < .000.

^S^ = Scheffé.

IM = intellectual motivation. SM = social motivation. CM = competence mastery. SA = stimulus avoidance.

One-way ANOVA was also used to examine the differences among the four leisure motivations based on income, and no significant differences were found. Post hoc analysis also indicated that there were no significant differences among income groups. Income did not seem to have any influence on leisure participation and leisure motivation. Table 9 shows the mean and SD.

**Table 9:** Differences among the four dimensions of LMS by income.

	¥1,000 or less		¥1,001–3,000		¥3,001–5,000		¥5,001 or More	
	M	SD	M	SD	M	SD	M	SD
IM	30.07	5.78	29.05	4.84	28.73	5.47	28.23	5.31
SM	27.64	6.14	28.19	4.21	27.84	5.15	27.55	5.72
CM	29.95	5.61	28.74	4.58	28.47	5.07	28.28	5.34
SA	31.28	4.80	30.77	4.83	30.74	4.69	30.09	4.76

Note. N = 453.

*p < .05.

** *p* < .01.

***p < .000.

IM = intellectual motivation. SM = social motivation. CM = competence mastery. SA = stimulus avoidance.

Table 10 shows the crosstab for gender and marital status. Among the female residents, 49.2% (125) were single, and only 0.8% (2) were divorced. Among the residents who were married with children, 52% (26) were male, and 48% (24) were female. A comparison of the residents who were single and those who were married with no children, the results indicated that gender may not be an influencing factor of marital status.

**Table 10:** Crosstab for gender and marital status.

Gender		Marital Status				Total
		Single	Married with Children	Married with No Children	Separated & Divorced	
Male	Count	89	26	82	2	199
	% Within Gender	44.7%	13.1%	41.2%	1.0%	100%
	% Within Marital Status	41.6%	52.0%	44.3%	50.0%	43.9%
	% of Total	19.6%	5.7%	18.1%	0.4%	43.9%
Female	Count	125	24	103	2	254
	% Within Gender	49.2%	9.4%	40.6%	0.8%	100%
	% Within Marital Status	58.4%	48.0%	55.7%	50%	56.1%
	% of Total	27.6%	5.3%	22.7%	0.4%	56.1%
Total	Count	214	50	185	4	453
	% Within Gender	47.2%	11.0%	40.8%	0.9%	100%
	% Within Marital Status	100%	100%	100%	100%	100%
	% of Total	47.2%	11.0%	40.8%	0.9%	100%

Note. N = 453.

Pearson correlations were used to explore the leisure activities that had the strongest relationships with leisure motivation. Table 11 showed the coefficients of the top five activities toward leisure motivation. Among the top five activities, traveling and taking a walk in the park were the strongest predictors for social motivation, and drinking tea and chatting was the strongest predictor for competence mastery. It is only statistically significant relationship.

**Table 11:** Correlation coefficients of the top five leisure activities for leisure motivation.

	IM		SM		CM		SA	
	r	p	r	p	r	p	r	p
internet surfing	.02	.74	.04	.40	.06	.21	.03	.58
drinking tea and chatting	-.08	.07	-.07	.12	-.14**	.00	.08	.10
traveling	.08	.07	.11*	.02	.08	.09	.08	.09
watching films, TV, & cartoons	.01	.80	-.07	.15	-.08	.09	-.07	.12
taking a walk in the park	.00	.97	-.10*	.03	-.04	.39	.06	.20

Note. N = 453. Multiple R’s for IM, SM, CM, and SA are .12, .18, .19, and .16, respectively. Dependent variable: Leisure Motivation. IM = intellectual motivation. SM = social motivation. CM = competence mastery. SA = stimulus avoidance.

Leisure time, leisure frequency, and leisure spending were transformed into dichotomous variables to find the strongest predictor for leisure motivation by using Pearson correlations. The responses that indicated a leisure time of less than 14 hours a week were categorized as “0”, and the responses that indicated a leisure time of more than 14 hours a week were categorized as “1”. The responses that indicated a leisure frequency of less than 5 times a week were categorized as “0”, and the responses that indicated a leisure frequency of more than 7 times a week were categorized as “1”. The responses that indicated a leisure expense of less than ¥199 a week were categorized as “0”, and the responses that indicated a leisure expense of more than ¥200 a week were categorized as “1”. Table 12 shows the coefficients for leisure time, leisure frequency, and leisure spending for leisure motivation. The results showed that leisure frequency was the strongest predictor for social motivation, and leisure spending had a significant relationship with social motivation. It is only statistically significant relationship.

**Table 12:** Correlation coefficients of leisure time, leisure frequency, and leisure spending for leisure motivation.

	IM		SM		CM		SA	
	r	p	r	p	r	p	r	p
leisure time	-.02	.71	-.06	.18	-.05	.32	-.06	.20
leisure frequency	-.08	.09	-.13**	.00	-.03	.56	-.07	.11
leisure spending	.07	.16	.11*	.01	.09	.07	.04	.45

Note. N = 453. Multiple R’s for IM, SM, CM, and SA are .11, .18, .10, and .09, respectively. Dependent variable: Leisure Motivation. IM = intellectual motivation. SM = social motivation. CM = competence mastery. SA = stimulus avoidance.

## Discussion

The purpose of this study is to investigate the leisure activities and leisure motivations of the residents of Hangzhou, China. Demographic variables (gender, age, marital status, education level, and income) were also examined.

### Status quo of residents’ leisure in Hangzhou

The top three favorite activities of residents are internet surfing (56.1%), drinking tea and chatting (46.1%), and traveling (44.4%). The results show that with the rapid technology development as well as the social and economic progress over the past twenty years in Hangzhou, the residents’ lives in terms of leisure have changed substantially. Internet surfing and other high-tech leisure activities account for a much higher proportion of residents’ leisure participation, while traditional leisure such as playing poker and mahjong (7.7%) and hobbies (e.g., painting, reading, photography and collections) (10.2%) are less frequent in residents currently. These results are consistent with Su’s conclusion [17], in which college students’ top three favorite leisure activities are internet surfing, cell phone text communication, and sports.

Hangzhou is a famous city for its natural beauty and is always crowned as the “Capital of Tea”, “Home of Silk”, and “Paradise on Earth”. Hangzhou is best known as the origin of the Tea of Longjing, a notable green tea. Teahouses can be seen everywhere in Hangzhou. Therefore, it is not surprising that drinking tea and chatting (46.1%) are some of the residents’ favorite leisure activities. In addition to internet surfing (56.1%) and drinking tea and chatting (46.1%), traveling (44.4%) is also a favorite leisure activity of residents. Hangzhou has rich tourism resources, including West Lake, Lingyin Temple, and Xixi National Wetland Park. Traveling is essential for the residents of Hangzhou. Among the top five activities, traveling was the strongest predictor for social motivation, while drinking tea and chatting was the the strongest predictor for competence mastery.

### Status quo of residents’ leisure motivation in Hangzhou

As shown in Table 4, overall, residents’ leisure motivations, including intellectual, social, competence mastery, and stimulus avoidance, all had higher levels. Female and male residents both had the highest scores in competence mastery and stimulus avoidance, but female residents had a much stronger desire to pursue peaceful and calm leisure items than did male residents. The results indicated that there were no significant differences among leisure motivations by age and income. Age and income did not seem to have any influence on leisure participation and leisure motivation at all.

There were significant differences in intellectual motivation by marital status. Residents who were separated or divorced had the highest scores on intellectual motivation. The findings indicated that single residents were more likely to show intellectual motivation. Thus, partnership played an important role in intellectual motivation. In addition, education seemed to affect residents’ leisure motivations. Residents who attained higher education levels may be better able to financially maintain their daily lives. The results showed that residents with high education levels had the lowest mean scores on social motivation.

## Summarization

This research makes several significant contributions to the literature on leisure. First, by focusing on leisure activities and leisure motivations of the residents of China, the researchers determined the reasons why residents choose to engage or disengage in different activities and how residents’ gender, age, marital status, education level and income relate to their leisure motivations. Second, the current findings provide a foundation for further in-depth study of leisure motivation. The study of residents’ leisure should involve their own history and culture. Finally, these findings provide beneficial information for investigating Chinese leisure motivations.

Because of an oversight, career, which should be another important factor that affects leisure behavior, was not measured in this research. Many existing researches on leisure motivation involved the career factor. The shortened version of the Leisure Motivation Scale was used in a sample of 1,127 British vacationers [18]. Starzyk et al. used the Leisure Motivation Scale to study leisure motivation and psychosocial adjustment among young offenders and high school students [19]. Meanwhile, the relationship among leisure satisfaction, leisure motivation, and perceived freedom in leisure was examined in 84 male offenders [20], and young offenders and high school students differed significantly in terms of personality but not leisure motivation [21]. The results showed that residents’ careers should be considered to help explain leisure motivations. Furthermore, additional career types should be examined in developing countries in future work. In addition, when Walker investigated Canadian and Mainland Chinese students’ leisure behaviors, he evaluated the role of self-construal, which was used as an intervening variable between culture and motivation [10]. Self-construal and self-determination theory (SDT) should be taken into account to examine the residents’ leisure motivations in future research.

## Supporting information

S1 Appendix Demographic survey. (DOCX)

S2 Appendix Leisure participation involvement. (DOCX)

S3 Appendix Leisure Motivation Scale. (DOCX)
