# Supplementary material for: Leisure activities and leisure motivations of Chinese residents
Source: PLoS One. 2018 Nov 1;13(11):e0206740. doi: 10.1371/journal.pone.0206740 (PMC6211730; doi:10.1371/journal.pone.0206740)
Supplement: S1 Appendix — (DOCX) [file pone.0206740.s001.docx]

**Demographic Survey**

1. What is your gender: (1) ________Male (2)___________Female

2. What is your age:

(1) ________ 18^_^ (2) ________18-24 (3) ________ 25-35

(4) ________36-45 (5) ________46-55 (6) ________55+

3. What is your educational level attained?

(1) ________Senior School or Lower

(2) ________Junior College

(3) ________Undergraduate College

(4) ________Master’s/PhD

4. What is your marital status?

(1) ________Single

(2) ________Married with Children

(3) ________Married with no Children

(4) ________Separated & Divorced

5. What is your monthly income?

(1) ________¥1,000 or less

(2) ________¥1,001-3,000

(3) ________¥3,001-5,000

(4) ________¥5,001 or More
