# Supplementary material for: Leisure activities and leisure motivations of Chinese residents
Source: PLoS One. 2018 Nov 1;13(11):e0206740. doi: 10.1371/journal.pone.0206740 (PMC6211730; doi:10.1371/journal.pone.0206740)
Supplement: S2 Appendix — (DOCX) [file pone.0206740.s002.docx]

**Leisure Participation Involvement**

**1.** What leisure activities do you enjoy participating in frequently? (Choose three items)

(a) internet surfing (b) drinking tea and chatting (c) traveling

(d) watching films, TV, and cartoons (e) taking a walk in the park

(f) visiting relatives and friends (g) sports

(h) visiting (museum or celebrity house) (i) hobbies (e.g., painting, reading,

photography and collections) (j) playing poker and mahjong

(k) taking care of plants and pets (l) alcohol consumption

(m) theatre (n) skincare, decorating the house (o) others

2. How many hours do you spend on leisure each week?

(a) 7 hours or less

(b) 7-14 hours

(c) 14-21 hours

(d) 21 hours or more

3. On how many occasions do you participate in leisure each week?

(a) three times or less

(b) 3-5 times

(c) 6-7 times

(d) 7 times or more

4. What are your weekly expenses on leisure (RMB)?

(a) ¥100 or below

(b) ¥100-199

(c) ¥200-499

(d) ¥500 or more
