# Supplementary material for: Leisure activities and leisure motivations of Chinese residents
Source: PLoS One. 2018 Nov 1;13(11):e0206740. doi: 10.1371/journal.pone.0206740 (PMC6211730; doi:10.1371/journal.pone.0206740)
Supplement: S3 Appendix — (DOCX) [file pone.0206740.s003.docx]

**Leisure Motivation Scale (English)**

Please indicate how true fir each of the following statement. Read each statement and

then circle the appropriate answer. If you strongly disagree the statement, please circle

" 1 " . If you strongly agree the statement, please circle "5". If you are in between, please circle the number which describes how true the statement is for you.

|  | Strongly  disagree |  |  |  | Strongly  agree |
| --- | --- | --- | --- | --- | --- |
| 1. to explore new ideas | 1 | 2 | 3 | 4 | 5 |
| 2. to satisfy my curiosity | 1 | 2 | 3 | 4 | 5 |
| 3. to learn about things around me | 1 | 2 | 3 | 4 | 5 |
| 4. to expand my knowledge | 1 | 2 | 3 | 4 | 5 |
| 5. to learn about myself | 1 | 2 | 3 | 4 | 5 |
| 6. to discover new things | 1 | 2 | 3 | 4 | 5 |
| 7. to be creative | 1 | 2 | 3 | 4 | 5 |
| 8. to use my imagination | 1 | 2 | 3 | 4 | 5 |
| 9. to interact with others | 1 | 2 | 3 | 4 | 5 |
| 10. to build friendship with others | 1 | 2 | 3 | 4 | 5 |
| 11. to gain a feeling of belonging | 1 | 2 | 3 | 4 | 5 |
| 12. to socially competent and skillful | 1 | 2 | 3 | 4 | 5 |
| 13. to meet new and different people | 1 | 2 | 3 | 4 | 5 |
| 14. to reveal my thoughts, feelings, or  physical skills to others | 1 | 2 | 3 | 4 | 5 |
| 15. to develop close friends | 1 | 2 | 3 | 4 | 5 |
| 16. to gain others respect | 1 | 2 | 3 | 4 | 5 |
| 17. to challenge my ability | 1 | 2 | 3 | 4 | 5 |
| 18. to be active | 1 | 2 | 3 | 4 | 5 |
| 19. to improve my skill and ability in  doing them | 1 | 2 | 3 | 4 | 5 |
| 20. to be good in doing them | 1 | 2 | 3 | 4 | 5 |
| 21. to keep in shape physically | 1 | 2 | 3 | 4 | 5 |
| 22. to use my physical abilities | 1 | 2 | 3 | 4 | 5 |
| 23. to develop physical skills and abilities | 1 | 2 | 3 | 4 | 5 |
| 24. to develop physical fitness | 1 | 2 | 3 | 4 | 5 |
| 25. to slow down | 1 | 2 | 3 | 4 | 5 |
| 26. to rest | 1 | 2 | 3 | 4 | 5 |
| 27. to relax mentally | 1 | 2 | 3 | 4 | 5 |
| 28. to avoid the hustle and bustle of daily  activities | 1 | 2 | 3 | 4 | 5 |
| 29. to relax physically | 1 | 2 | 3 | 4 | 5 |
| 30. because I sometimes like to be alone | 1 | 2 | 3 | 4 | 5 |
| 31. to unstructured my time | 1 | 2 | 3 | 4 | 5 |
| 32. to release stress and tension | 1 | 2 | 3 | 4 | 5 |

`
